# Leveraging shortest dependency paths in low-resource biomedical relation extraction

**DOI:** 10.1186/s12911-024-02592-2

**Published:** 2024-07-24

**Authors:** Saman Enayati, Slobodan Vucetic

**Affiliations:** Department of Computer and Information Sciences, Temple University, 1925 N. 12th Street, Suite 304, Philadelphia, PA 19122 USA

**Keywords:** Natural language processing, Relation extraction, Low-resource, Shortest dependency path, BERT

## Abstract

**Background:**

Biomedical Relation Extraction (RE) is essential for uncovering complex relationships between biomedical entities within text. However, training RE classifiers is challenging in low-resource biomedical applications with few labeled examples.

**Methods:**

We explore the potential of Shortest Dependency Paths (SDPs) to aid biomedical RE, especially in situations with limited labeled examples. In this study, we suggest various approaches to employ SDPs when creating word and sentence representations under supervised, semi-supervised, and in-context-learning settings.

**Results:**

Through experiments on three benchmark biomedical text datasets, we find that incorporating SDP-based representations enhances the performance of RE classifiers. The improvement is especially notable when working with small amounts of labeled data.

**Conclusion:**

SDPs offer valuable insights into the complex sentence structure found in many biomedical text passages. Our study introduces several straightforward techniques that, as demonstrated experimentally, effectively enhance the accuracy of RE classifiers.

## Introduction

Biomedical Relation Extraction (RE) plays a pivotal role in structuring unstructured medical texts, enabling the construction of knowledge graphs [1, 2] and the extraction of complex relationships between biomedical entities such as drugs, proteins, and genes [3–5]. Effective RE aids in the discovery of new drug interactions and biological pathways, critical for advancing medical research and clinical decision-making.

Despite advancements through supervised RE methods [6–8], their efficacy is often limited by the scarcity of labeled biomedical data. Several approaches, such as weak supervision [9] and semi-supervised learning (SSL) [10], have been developed to address the challenges of limited training data through leveraging unlabeled data. More recently, in-context learning techniques using Large Language Models (LLMs) have emerged, requiring significantly less labeled data [11, 12].

Weak supervision, for instance, utilizes heuristics, rules, or distant supervision to generate noisy labels for unlabeled data, a technique pioneered by [9]. Although this method enhances the volume of trainable data, it also introduces label noise. Other strategies, such as those developed by [13, 14], employ linguistic patterns like SDP (Shortest Dependency Path) tokens or frequent phrases to formulate labeling rules, reducing the need for manual annotation yet increasing the hidden costs of rule derivation.

SSL utilizes both a limited pool of labeled data and a larger volume of unlabeled data to enhance learning models [10]. Common SSL strategies such as self-training rely heavily on predictors that impute labels on unlabeled examples, which are then used to retrain the models [15]. Variants of self-training include dual training, where a secondary predictor retrieves relevant unlabeled instances [16], and Gradient Imitation Reinforcement Learning (GIRL), which optimizes the correlation between gradients of labeled and unlabeled data to enhance performance [17].

The challenge in self-training is limited labeled data leads to low-quality label imputations, as the reliance on the predictor impedes learning a powerful model. Likewise, graph-based SSL methods, such as label propagation, utilize lexical and syntactic features to propagate labels among closely situated data points [18, 19]. However, determining the correct distance metric and neighborhood thresholds necessary for identifying relevant relational patterns within data remains an open research question. This issue also affects in-context learning, where selecting relevant examples from a constrained dataset for task demonstrations also requires precise distance metrics.

**Fig. 1:**
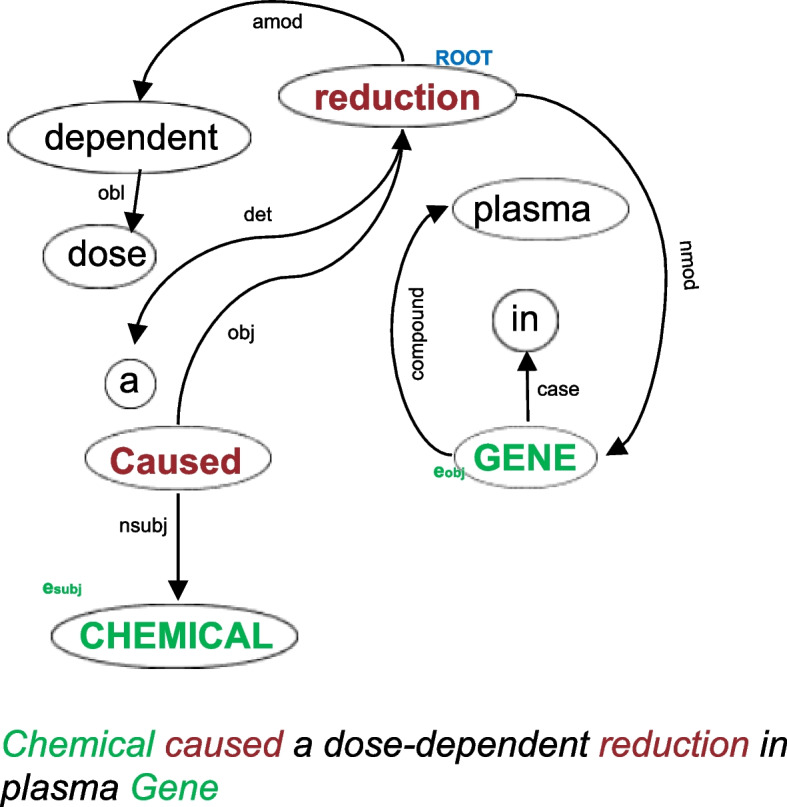
A dependency parse tree on a biomedical sentence and its shortest dependency path (SDP) tokens (shown in red) between subject (CHEMICAL) and object (GENE) entities

This study aims to address two pivotal questions: What is an effective representation for defining a suitable distance metric in low-resource settings, and how can we reduce the model’s dependence on imputed labels to boost RE accuracy in SSL scenario? To tackle the first question, we propose employing the SDP between entities to process the encoder output and compute the *SDP representation* for RE. SDP, derived from dependency parse trees, identifies the minimal syntactic dependencies essential for connecting entities, providing valuable hints about their relationships [20–22] (see Fig. 1).

For the second question, we advocate using a nearest neighbor approach instead of relying solely on model-based imputations. This method carefully propagates labels to nearby data points based on a specifically defined SDP-based distance metric, thereby allowing for the integration of soft labeling techniques that account for the uncertainty and noise in label imputation.

By addressing these research questions and conducting extensive experiments across three biomedical RE benchmarks, we aim to develop a versatile strategy compatible with any standard RE architecture and SSL algorithm. This approach is designed to deliver accurate results in various low-resource environments, encompassing supervised, SSL, and in-context learning scenarios. In summary, the contributions of this paper are:

- We propose utilizing SDP to calculate SDP representation of entity pairs for RE, improving accuracy in various low-resource settings such as supervised learning and in-context learning.
- We use SDP representation to calculate SDP-based distance metric between RE examples. We use this distance metric to support two types of SSL algorithms. We experimentally evaluate on biomedical text the usefulness on the distance metric.
- Our extensive experiments on three key biomedical RE benchmarks confirm the efficiency of our proposed method in low-resource settings.

## Background and task formulation

In this section, we introduce the relevant concepts and formally define the RE task.

**Shortest dependency path (SDP).** Let $\mathcal {T}$ be a dependency parse tree corresponding to sequence *s* representing the syntactical relationship between words in a sentence. In a dependency parse tree, words are represented as nodes, and the relationships between words are represented as directed edges. Given a pair of entities $(e_s, e_o)$, SDP is defined as the minimum set of tokens that can be reached from $e_s$ to $e_o$ through the dependency tree $\mathcal {T}$. Figure 1 shows an example of a parse tree, where the extracted SDP tokens between a pair of entities (*Chemical* and *Gene*) are highlighted in red. We need to highlight that we don’t consider the relation dependency between words in this study.

**Relation extraction (RE).** Given sentence $s=(w_1,w_2,...,w_n)$, a subject entity $e_{s}$, and an object entity $e_{o}$, the RE task is to predict the relation label $r \in \mathcal {R}$ of triple $x=(s,e_s,e_o)$, where $\mathcal {R}$ is a union of a predefined set of relation types and *None*, referring to no relation or other type of relation.

**Semi-supervised RE.** This approach utilizes a small set of labeled examples $\mathcal {D}_L=\{(x_i,r_i)\}_{i=1}^{N_l}$ and a larger set of unlabeled examples $\mathcal {D}_U=\{(x_j)\}_{j=1}^{N_u}$ to train a classifier model $f_{\theta }$. The model aims to fit the labeled data while also leveraging the unlabeled data to improve overall accuracy.

In the following subsections, we refer to $x=(s,e_s,e_o)$ as an RE example and *r* as the RE label. In addition, we assume the entity mentions can be identified using external tools and the set of relation types $\mathcal {R}$ is defined.

## Methodology

### SDP representation

In this section, we propose the SDP representation for RE. RE is defined as a text classification problem. As an encoder, we utilize the BERT neural network architecture [23]. BERT takes a sentence of tokens as input and produces embedding vectors for the entire sentence and each individual token, denoted as $H:[h_{cls},h_1,...,h_n] = \text {BERT}(w_1,...,w_n)$, where $w_i$ represents the *i*-th token in the sentence, $h_i$ is its embedding, and $h_{cls}$ is the sentence embedding.

**Fig. 2:**
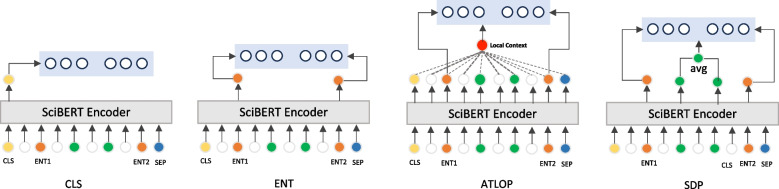
Comparison between different representations to fine-tune a RE using a linear layer on top of an encoder. Orange corresponds to the entity representation. Green corresponds to the SDP tokens between entities

This sequence of vector embeddings can be used in multiple ways as an input to the classification head. As illustrated in Fig. 2, we first present several baseline approaches for using a sequence of embedding vectors. Then, we propose how to calculate the SDP embedding.

**CLS:** BERT CLS embedding is used as an input to the RE decoder. This representation has been commonly used for many downstream text classification tasks, including RE [6, 7]. We denote the dimension of this token as *L*, which corresponds to the length of $h_{cls}$.

1 $$\begin{aligned} \text {CLS}_{\text {rep}} = h_{cls} \end{aligned}$$

**ENT:** Followed by the best setup in [24], we concatenate embeddings of the two entity tokens. If an entity consists of multiple tokens we only consider the first token.

2 $$\begin{aligned} \text {ENT}_{\text {rep}} = h_s \oplus h_o \end{aligned}$$

where $\oplus$ denotes the concatenation. The dimension of this representation as 2*L* which corresponds to $h_{s}$ and $h_{o}$.

**ATLOP:** Based on [8], we combine the entity embeddings using a vector called the local context. This vector contains pertinent information related to both entities. Let’s consider $A_s$ and $A_o$ as the self-attention matrices for entities $e_s$ and $e_o$ from the last layer in BERT, where $A \in R^{H\times l \times l}$. $A_{ijk}$ represents attention from token j to token k in the $i_{th}$ attention head, while $A_{i}^E \in R^{H \times l}$ denotes attention from the $i_{th}$ entity to all tokens. We locate the local context that is important to both $e_s$ and $e_o$ by multiplying their entity-level attentions, and obtain the localized context embedding *c*(*s*, *o*) by:

3 $$\begin{aligned} A^{(s,o)}{} & {} = A_s^E \odot A_o^E, \nonumber \\ q^{(s,o)}{} & {} = \sum \limits _{i=1}^{H} A_{i}^{(s,o)}, \nonumber \\ a^{(s,o)}{} & {} = q^{(s,o)} / 1^{\intercal } q^{(s,o)}, \nonumber \\ c^{(s,o)}{} & {} = H^{\intercal } a^{(s,o)} \end{aligned}$$

Where *H* is the contextual embedding derived from BERT. To construct the final embedding for an instance, we concatenate the local context embedding with the embeddings of the entity pair:

4 $$\begin{aligned} \text {ATLOP}_{\text {rep}} = h_s \oplus c^{(s,o)} \oplus h_o \end{aligned}$$

The resulting representation has length $3 \times L$.

**SDP**_**rep**_**:** Our main contribution is the use of SDP to enrich the embeddings by focusing on syntactic paths that are most indicative of relations. Biomedical sentences use complex terminology can be quite long, but they follow a relatively structured grammar. We hypothesize that the SDP between pairs of entities in biomedical documents is very likey to contain key information in revealing the relation type. To this end, we empploy SDP to direct our attention mechanism within BERT encoder. By averaging the embeddings of the tokens that constitute the SDP and concatenating these with the embeddings of the starting and ending tokens of the entities involved, we form a meaningful representation which can be formulated as follow:

5 $$\begin{aligned} SDP_{rep} = h_s \oplus \left(\frac{1}{|SDP|} \sum \limits _{w_i \in SDP} h_i \right) \oplus h_o, \end{aligned}$$

where |*SDP*| denotes the number of SDP tokens. The dimension of SDP representation is $3 \times L$, which blends syntactic precision with semantic richness.

In the RE model, the decoder is a single classification layer $W \in R^{K \times L^*}$ where $L^*$ is the dimension of the input representation ($L^*=3L$ for SDP representation) and K is the number of relation types. Importantly, this decoder can be replaced with any off-the-shelf RE architecture and is placed on top of the processed encoder representation. In addition, the SDP representation can be integrated separately to define a distance metric such as in in-context learning or retrieval scenarios, enhancing the model’s applicability to a broader range of tasks that require a nuanced understanding of entity relationships. The classification of the RE task given a representation is performed by:

6 $$\begin{aligned} p(r|x) = \text {softmax}\left(V_x W^T\right) \end{aligned}$$

where $V_x$ is the output vector from the last layer of the encoder corresponding to the SDP-enhanced representation for input *x*, and *W* represents the weight matrix of the classification layer. The loss for training is computed as the cross-entropy between *p*(*r*|*x*) and the true relation labels.

### SDP in semi-supervised RE

This section explores the integration of the Shortest Dependency Path (SDP) with nearest neighbor-based label propagation in an extreme low-resource SSL setting. Our approach is designed to effectively utilize a minimal set of labeled data points, thereby eliminating the need for a large labeled for training and validation set.

Our primary goal is to leverage the SDP representation in conjunction with nearest neighbor techniques to compute distances between RE examples. This enables precise label propagation and reduces reliance on predictors for generating pseudo-labeled data.

We developed an SSL approach that combines graph-based SSL and self-training. Similar to graph-based SSL, our algorithm propagates labels to closely neighboring unlabeled data Unlike traditional graph-based methods, it meticulously restricts label propagation to only the nearest neighbors rather all than unlabeled data [25]. This constraint is essential in low-resource settings, as it minimizes the noise introduced by including broader sets of unlabeled data and preserves the influence of the scarce labeled data available in the training process. From the self-training perspective, while our approach utilizes the predictor to extract representations, it does not rely on it to generate pseudo-labels. This is because it is challenging to develope a reliable and unbiased predictor with limited labeled data. Instead, we utilize these representations to compute soft labels, which are determined based on the proximity of unlabeled RE examples to their labeled counterparts. This technique indirectly employs the predictor, enhancing the label quality without the direct use of potentially biased pseudo-labels. Prior research shows that soft labels result in a more robust classifier [26] by informing the training process about the quality of imputed labels. To compute soft labels for unlabeled examples, we follow the steps below:

- Find the nearest neighbor of the unlabeled example among the labeled examples.
- Calculate the cosine similarity(*d*) between the unlabeled example and its top-k nearest neighbors (labeled data).
- Aggregate the cosine similarities for each class type
- Compute the soft labels for each class type by normalizing the aggregated similarities using softmax.

To optimize the RE model on soft labels, we use Noise Aware Cross Entropy loss (Eq. 7), which is computed for each class separately, and then the losses are summed together,

7 $$\begin{aligned} \mathcal {L} = \sum \limits _{i=1}^N \sum \limits _{c=1}^K y_{i,c} \log {\hat{y}_{i,c}}, \end{aligned}$$

where *N* is the total number of examples, and $y_{i,c}$ and $\hat{y}_{i,c}$ represent the ground truth and predicted soft label for example *i* and class *c*. The algorithm is executed iteratively, with each cycle incorporating a limited number of additional soft labels. We specify a fractional amount of unlabeled data as validation set and monitor the predictor’s fluctuations to determine convergence. Convergence is achieved when the prediction variation on a validation set is less than 5% between iterations, or when the maximum number of iterations is reached. It is important to note that our algorithm does not rely on ground truth validation data, using instead the stability of predictions as a stopping criterion.

This semi-supervised approach, centered on the strategic use of SDP and soft labeling along with nearest neighbor-based propagation, is designed to enhance the efficiency of RE models in SSL settings constrained by extreme limited labeled data.

## Experiments

### Dataset

We evaluate the effectiveness of our method on three public biomedical relation extraction datasets retrieved from PubMed database. The statistics of these datasets are shown in Table 1. 1) **ChemProt** [27] consists of 1,820 PubMed abstracts with chemical-protein interactions annotated by domain experts and was used in the BioCreative VI text mining chemical-protein interactions shared task. 2) **DDI** [28] contains MedLine abstracts on drug-drug interactions as well as documents describing drug-drug interactions from the DrugBank database. 3) **PPI** [29] utilizes AIMed corpus to automatically extract interaction relations of protein-protein pairs affected by genetic mutations.

**Table 1:** Statistics of each dataset

Dataset	Train	Validation	Test	#Relations
ChemProt	18k	11.2k	15.7k	6
DDI	22k	5.5k	5.7k	5
PPI	5.2k	521	583	2

### Compared methods

To perform experiments, we first compared our SDP-based finetuning strategy with several supervised RE baselines. Then, we adopted the best-performing baseline as the RE classifier model to explore the impact of SDP in SSL baselines and in-context-learning. In all the experiments, we applied SciBERT [30] as the encoder. We performed all the experiments under a very limited budget for labeled data and abundant unlabeled data. We denote $\text {SUP-RE}_{sdp}$ and $\text {SSL-RE}_{sdp}$ as supervised and semi-supervised variants of SDP in the remaining subsections.

**Supervised baseline methods:** The goal is to compare the performance of different RE architectures (as discussed in “SDP representation” section) in a supervised setting and show that fine-tuning using $\text {SUP-RE}_{sdp}$ achieves a better performance compared to the existing approaches. These approaches are *CLS*, *ENT*, *ATLOP*. This experiment utilizes limited labeled data to explore the best-performing RE model (in our case is 500).

**Semi-supervised baseline methods:** The goal of this experiment is to compare the superior performance of $\text {SSL-RE}_{sdp}$ with predictor-based SSL. To ensure fair comparison with $\text {SSL-RE}_{sdp}$, we used the best-supervised baseline from Table 2, which was $\text {SUP-RE}_{sdp}$, as RE model. We applied the following SSL methods on $\mathcal {D}_{L} \cup \mathcal {D}_{U}$:

(1) *Label Propagation* [25], which is a graph-based algorithm that iteratively updates the label probability in $\mathcal {D}_{U}$ by matrix multiplication (*TR*, where *T* is a $n \times n$ weighted adjacency matrix (pairwise relations between labeled and unlabeled data) and *R* is $n \times C$ class probability matrix). (2) *Self-Training* [15], which iteratively expands $\mathcal {D}_{L}$ by using the most confident (above $\tau$) predictor’s prediction among $\mathcal {D}_{U}$. (3) *DualRE* [16], which is a dual training algorithm that utilizes a learning-to-rank model as a dual module to retrieve the relevant instances from $\mathcal {D}_{U}$ for a given relation. (4) *RE-Ensemble*, which replaces the dual module in *DualRE* [16] with the same predictor in the primal module, with a different random initialization. *RE-Ensemble* imputes the labels based on the agreement of the two modules.

We also provide *SUP-RE*_sdp_ as a supervised baseline, which can also serve as a few-shot baseline since it is only trained on limited labeled data without access to unlabeled data. In addition, we report *SSL-RE*_sdp_*-1-Iter*, which is the same as $\text {SSL-RE}_{sdp}$, but only uses one iteration to perform imputation.

### Experimental setting

**Implementation:** We implemented all the baselines using Pytorch. For *DualRE* and *RE-Ensemble* [16], we replaced the Position-aware Recurrent Neural Network that was used originally in [31] with $\text {SUP-RE}_{sdp}$. The source code for these baselines can be found here^1^. In addition, we used the code provided by [32] to apply label propagation algorithm.

**Training details:** We adopted SciBERT as the encoder for all the experiments and update all the parameters. For supervised finetuning, we add one linear layer followed by softmax to perform classification. We use the following set of hyper-parameters as suggested in [23]:

- Transformer Architecture: 12 Layers, 768 hidden dimension, 6 heads
- Learning rate: 3e-5
- Weight initialization: SciBERT-base
- Batch size: 32
- Optimizer: adam
- Training epochs: 5
- Maximum sequence length: 200

We used 1 GPU, Tesla V100-SXM2, for training. We applied SciSpacy [33] dependency parser to our corpus to retrieve the SDP tokens for an entity pair.

For SSL experiments, we kept the same hyper-parameters. We impute labels to the top-5 unlabeled data. A similar strategy is applied to retrieve labeled examples for each unlabeled data to compute soft-labels.

In *Self-Training*, since we use the RE model to provide predictions on unlabeled data, we set the threshold for the most likely class to be above 0.90. However, since the majority of the predictions were overconfident based on the validation results, resulting in imputing noisy labels, we only select the top 100 in the augmented set. In *Label Propagation* implementation based on [32], we chose KNN as kernel function, and set the *K* to 5, which specifies the number of closest labeled instances to include in the label propagation process for each unlabeled instance. For *DualRE* and *RE-Ensemble*, we followed the default hyperparameters mentioned in [16]. We only leveraged 50% of $\mathcal {D}_{U}$, and used the default confidence thresholds $\alpha =0.5$ and $\beta =2$ predictor and retrieval modules, respectively. We applied the same convergence criteria as in $\text {SSL-RE}_{\text {sdp}}$ for self-training and dual training.

**Evaluation metric:** Following the previous work in RE [14, 16], we report micro-F1 as the most important evaluation metric. It provides an evaluation of the model’s ability to simultaneously capture precision and recall across all classes. We ignored correct predictions of *None* in micro score calculation.

## Results

We conducted each experiment over three different independent sets of labeled data and reported the mean performance.

### Comparison with supervised baselines

Table 2 demonstrates the performance of different supervised RE architectures under 500 training budget. $\text {SUP-RE}_{sdp}$ approach achieves higher accuracy compared to the CLS, ENT, and ATLOP architectures. This can be attributed to the explicit guidance provided by SDP, which directs the predictor to focus on tokens relevant to the target label in biomedical settings where limited labeled data exists.

Among the approaches considered, the CLS representation exhibits the lowest performance. This could be due to the fact that it is sentence-level representation, having less relevant information for entities.

When compared to ATLOP, $\text {SUP-RE}_{sdp}$ appears to have slightly better F1 score across all datasets. This indicates that the local context pooling mechanism in ATLOP does not capture dependencies as accurately as $\text {SUP-RE}_{sdp}$. Furthermore, $\text {SUP-RE}_{sdp}$ slightly outperforms ENT-based fine-tuning on the DDI and ChemProt datasets, while delivering comparable performance on PPI.

To statistically validate the performance differences observed, a Repeated Measures ANOVA was conducted for each dataset. This analysis confirmed the significance of the observed variations in performance, with the *p*-value for DDI at $p=0.0030$, for ChemProt at $p=0.0028$, and for PPI at $p=0.0025$. The consistency of these statistically significant results supports the superior efficacy of the $\text {SUP-RE}_{sdp}$ approach across all examined datasets, reaffirming its selection for further analysis.

Considering the slightly better performance of $\text {SUP-RE}_{sdp}$, as shown in Table 2, and the statistical confirmation of its superiority through ANOVA testing, we have selected it as the RE model for the subsequent subsections. These findings emphasize the importance of methodological selection and highlight the benefit of leveraging SDP-guided approaches in low-resource settings for RE tasks.

**Table 2:** Performance of different RE finetuning architectures when trained using 500 labeled data. The average F1 performance is reported over 3 independent runs

	DDI			ChemProt			PPI		
	P	R	F1	P	R	F1	P	R	F1
CLS	0.24	**0.77**	0.36	0.16	0.52	0.24	0.37	0.82	0.51
ENT	0.28	0.74	0.40	**0.23**	0.62	0.33	0.45	**0.84**	0.59
ATLOP	0.28	0.74	0.41	**0.23**	0.60	0.33	0.45	0.82	0.58
**SUP-RE**_*sdp*_	**0.29**	**0.77**	**0.42**	**0.23**	**0.63**	**0.34**	**0.46**	0.80	**0.59**

#### Comparison with non-encoder baselines

This experiment evaluates our supervised relation extraction (RE) method, which integrates Shortest Dependency Paths (SDP) and BERT-based representations, against traditional non-encoder baselines utilizing SDP or dependency trees as graph kernels for relation extraction. The fundamental principle of these kernel methods is to assess the similarity between two sentences by examining how closely their structural patterns align. These kernels operate in conjunction with Support Vector Machines (SVM) to classify sentences. Our analysis focuses on the Protein-Protein Interaction (PPI) dataset due to the availability of extensive kernel method benchmarks. We adopted the experimental setup from [34] to ensure a consistent comparison with the kernel methods listed in Table 2 of their study. All experiments were conducted using 10-fold cross-validation on the full PPI dataset, corresponding to the AIMed results in Table 2 of [34]. Following their recommendations, we implemented entity blinding to prevent the influence of named entity recognition problems and to highlight entity locations to the classifier. Our results are compared with a range of kernel methods as detailed in Table 3:

**Edit distance kernel (edit)** [39]: This kernel calculates the similarity by measuring the edit distance between the shortest paths connecting protein names within a dependency tree. The similarity is determined by the minimum number of edit operations−deletions, insertions, or substitutions required to make one path identical to the other, normalized by the length of the longer path.

**Cosine similarity kernel (cosine)** [39]: This method computes the cosine similarity between vectors representing the shortest paths in a dependency parse tree between pairs of entities. It quantifies the number of common terms along these paths, adjusted for path length.

**All-paths graph kernel (APG)** [40]: APG considers all possible path lengths within the dependency parse and surface word sequence, assigning greater weight to paths closer to the shortest path between entities, thereby reflecting dependency proximity.

**k-band shortest path spectrum kernel (kBSPS)** [41]: This kernel extends the analysis beyond the shortest dependency path to include nodes within a specified k-band distance, enriching the contextual data for relationship extraction.

**Other kernels**: We further compare our method against kernels that utilize syntax tree representations of sentences, such as the Subtree kernel (ST) [35], Subset tree kernel (SST) [36], Partial tree kernel (PT) [37], and Spectrum tree kernel (SpT) [38].

**Table 3:** Comparative analysis of non-encoder based kernel methods using Shortest Dependency Paths (SDP) against our supervised method, which also utilizes SDP for representation. Performance metrics are evaluated using a 10-fold cross-validation on the PPI dataset

	P	R	F1
Syntax Tree Kernel			
ST [35]	40.3	25.5	30.9
SST [36]	42.6	19.4	26.2
PT [37]	39.2	31.9	34.6
SpT [38]	33.0	25.5	27.3
SDP kernel			
edit [39]	68.8	27.7	39.0
cosine [39]	43.6	39.4	40.9
APG [40]	62.9	48.9	54.7
kBSPS [41]	50.1	41.4	44.6
**SUP-RE**_*sdp*_ (Ours)	**81.2**	**78.0**	**79.4**

Table 3 showcases a comparative analysis between various non-encoder-based kernel methods and our SDP-based approach for relation extraction. Notably, our method, $\text {SUP-RE}_{sdp}$, significantly outperforms the other models in precision (P), recall (R), and F1 score, achieving 81.21% precision, 78.0% recall, and an F1 score of 79.4%. This demonstrates a marked improvement over traditional non-encoder methods like the APG kernel, which has the next highest F1 score of 54.7% but with a substantially lower recall. The kBSPS, while competitive to APG, still trails our method with an F1 score of 44.6%. The substantial lead in performance metrics highlights the effectiveness of integrating SDPs with BERT-based representations, providing evidence that our LLM-based representation using SPD captures complex semantic relationships more effectively than conventional kernel methods.

### Comparison with semi-supervised baselines

Table 4 shows the result of our approach compared to SSL baselines and few-shot supervised baseline ($\text {SUP-RE}_{sdp}$). According to the results, one can observe that $\text {SSL-RE}_{sdp}$ outperforms all of the baselines across all datasets, which demonstrates the effectiveness of our framework versus SSL baselines.

**Table 4:** The F1 comparison of $\text {SSL-RE}_{sdp}$ versus SSL baselines. $\text {SUP-RE}_{sdp}$ serves as the supervised lower bound. The lower/upper bound for F1 metrics is 0/1. We report the average performance across three independent runs

	50			100			200			500		
	P	R	F1	P	R	F1	P	R	F1	P	R	F1
**DDI**												
SUP-RE_*sdp*_	0.15	0.50	0.23	0.21	0.58	0.31	0.24	0.65	0.35	0.32	0.82	0.46
LabelPropagation	0.076	0.42	0.15	0.13	0.59	0.21	0.18	0.76	0.25	0.24	**0.88**	0.35
DualRE	0.22	0.38	0.27	0.27	0.44	0.34	0.29	0.62	0.40	**0.43**	0.76	0.54
REEnsemble	0.19	0.33	0.24	0.29	0.41	0.34	0.30	0.54	0.38	0.39	0.62	0.48
SelfTraining	0.21	**0.68**	0.31	0.21	0.70	0.33	0.25	**0.77**	0.37	0.31	0.85	0.45
SL-RE_*sdp *_(1-Iter)	0.17	0.63	0.26	0.21	**0.71**	0.33	0.22	0.76	0.34	0.26	0.84	0.40
SSL-RE_*sdp*_	**0.31**	0.51	**0.38**	**0.39**	0.65	**0.48**	**0.44**	0.71	**0.54**	**0.43**	0.78	**0.56**
**ChemProt**												
SUP-RE_*sdp*_	0.095	0.34	0.15	0.13	0.40	0.20	0.21	0.54	0.30	0.31	0.76	0.44
LabelPropagation	0.12	0.50	0.20	0.10	0.42	0.19	0.14	0.56	0.24	0.21	**0.83**	0.33
DualRE	0.15	0.26	0.13	0.19	0.49	0.27	0.23	0.46	0.30	0.36	0.69	0.48
REEnsemble	0.032	0.13	0.051	0.066	0.25	0.11	0.11	0.40	0.18	0.22	0.7	0.33
SelfTraining	0.15	**0.54**	0.23	0.17	0.55	0.26	0.23	0.61	0.33	0.33	0.76	0.46
SSL-RE_*sdp*_ (1-Iter)	0.13	0.39	0.20	0.17	0.52	0.26	0.24	**0.66**	0.35	0.30	0.76	0.43
SSL-RE_*sdp*_	**0.29**	0.44	**0.35**	**0.40**	**0.56**	**0.46**	**0.47**	**0.66**	**0.54**	**0.46**	0.72	**0.56**
**PPI**												
SUP-RE_*sdp*_	0.31	0.71	0.43	0.35	0.78	0.47	0.43	0.82	0.56	0.49	0.82	0.61
LabelPropagation	0.20	**0.91**	0.35	0.21	**0.99**	0.35	0.27	**0.95**	0.42	0.36	**0.88**	0.49
DualRE	0.38	0.52	0.28	0.33	0.78	0.46	0.41	0.64	0.47	0.61	0.64	**0.63**
REEnsemble	0.33	0.26	0.28	0.40	0.34	0.33	0.52	0.34	0.38	**0.67**	0.56	0.60
SelfTraining	0.38	0.74	0.43	0.34	0.78	0.47	0.38	0.87	0.53	0.48	0.84	0.61
SSL-RE_*sdp*_ (1-Iter)	0.31	0.69	0.43	0.35	0.74	0.47	0.40	0.83	0.54	0.38	0.86	0.53
SSL-RE_*sdp*_	**0.50**	0.54	**0.52**	**0.56**	0.66	**0.61**	**0.55**	0.77	**0.64**	0.39	0.84	0.53

$\text {SSL-RE}_{sdp}$ achieved consistent gain over *Label Propagation*, *Self-Training*, *DualRE*, *RE Ensemble* on all datasets and with different labeling budgets, except in PPI dataset trained on 500 budget where *DualRE* performed the best.

One can observe *Self-Training* and *DualRE* do not have stable performance due to reliance on the predictor to provide weak labels. For example, *Self-Training* outperforms *DualRE* in PPI dataset on [50, 100, 200] budgets, while underperforming *DualRE* on DDI and ChemProt occasionally. This provides evidence that predictor-based SSL models are sensitive to the performance of the RE model.

In addition, *Label Propagation* performed weaker than baselines which shows that its low quality of imputation damages the model’s performance.

It could be concluded that $\text {SSL-RE}_{sdp}$ benefits from iterative augmentation, after comparing to *SSL-RE*_sdp_*(1-ter)*, which only uses one pass of label imputation. In addition, it improves the performance over the supervised baseline by a significant margin in all the experiments.

#### Performance on different datasets

The marginal gain of $\text {SSL-RE}_{sdp}$ on PPI is smaller than on ChemProt and DDI in Table 4. This is because the size of PPI is $4.2 \times$ smaller than DDI and ChemProt. Therefore, the amount of unlabeled data may not be sufficient to identify the most similar neighbors. This can be observed on other baselines since they underperformed the supervised baseline on this dataset, except for *DualRE* trained on 500 budget.

#### Performance as a fraction of labeled data size

Based on the results in Table 4, $\text {SSL-RE}_{sdp}$ is the most advantageous when the labeled dataset is extremely small (around 100 - 500), which is common in Biomedical domain. In DDI, $\text {SSL-RE}_{sdp}$ can reduce the need for labeled data by up to $~5\times$, *SUP-RE*_sdp_ achieves 0.46 F1 when trained on $\mathcal {D}_L = 500$, while *SSL-RE*_sdp_[100] boosts *SUP-RE*_sdp_[500] performance by $4\%$ when using only $\mathcal {D}_L = 100$.

Similar outcome can be observed in ChemProt dataset. *SSL-RE*_sdp_[100] is $~2\times$ more accurate than *SUP-RE*_sdp_[200], while using $2\times$ less labeled data. *SSL-RE*_sdp_ is also more accurate than *SUP-RE*_sdp_ on PPI on 50, 100, and 200 budgets, reducing the labeling need by $4\times$, achieving 0.56 with *SUP-RE*_sdp_[200] and 0.52 with *SSL-RE*_sdp_[50].

Overall, one can observe that $\text {SSL-RE}_{sdp}$ is significantly beneficial when the cost of collecting labeled data is very high.

#### Statistical significance test

The t-test test^2^ for statistical significance has been used to find whether the difference between $\text {SSL-RE}_{sdp}$ and other SSL baselines are due random chance. Therefore, we define the null hypothesis as there is not a significant difference in the performance of $\text {SSL-RE}_{sdp}$ and other baselines. To this end, we use the final F1 scores from 3 independent runs across 4 labeling budgets to calculate *p*-value and t-statistics. We report the pvalue of our method compared to label propagation, self-training, RE-Ensemble, and dualRE in Table 5. The reported results reject the null hypothesis for all the baselines as they are all less than the significance level of 0.05, meaning our results are significantly better than baselines. This can be confirmed through t-statistic’s magnitude, since it is positive which indicates a higher difference between the average performance of $\text {SSL-RE}_{sdp}$ versus baselines and suggests stronger evidence against the null hypothesis.

**Table 5:** T-test analysis of $\text {SSL-RE}_{sdp}$ versus baselines

	T-statistic	*P*-value
LabelPropagation	9.5	3e-14
DualRE	4.8	1e-05
REEnsemble	6.8	3e-09
SelfTraining	4.9	6e-06

**Fig. 3:**
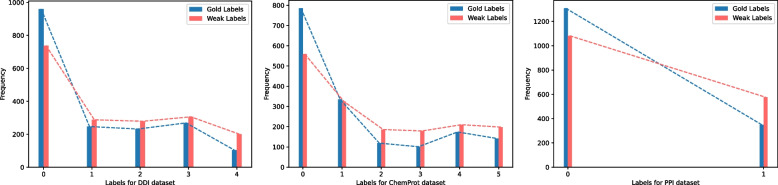
Comparing the distribution of imputed labels in the augmented examples (red bars) to their actual labels (blue bars) on DDI, ChemProt, and PPI dataset

#### Imputation bias analysis

To validate $\text {SSL-RE}_{sdp}$ could prevent any imputation bias (e.g. a certain RE type is overpredicted) due to label noise and enables quality weak labels, in Fig. 3, we represent gold label distribution with blue and weak label distribution with green. Technically, we have the ground truth labels available for all imputed weak labels. From Fig. 3, we observe that weak label distribution is close to the gold label distribution with less drift.

#### Qualitative analysis of $\text {SSL-RE}_{sdp}$ versus baselines

**Fig. 4:**
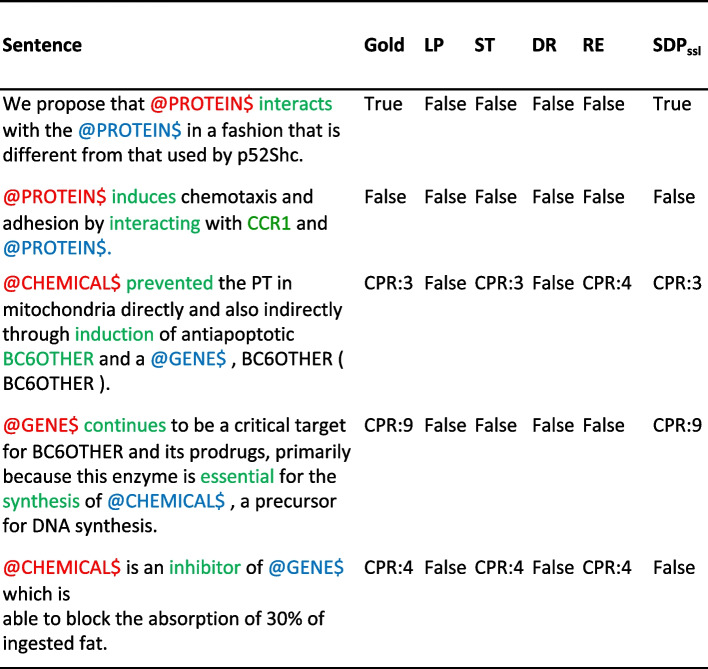
Qualitative Analysis of $\text {SSL-RE}_{sdp}$ vs baselines on PPI and ChemProt datasets. LP, ST, DR, RE are denoted as Label Propagation, Self-Training, Dual-RE, and RE-Ensemble

Figure 4 demonstrates few examples of the actual prediction of baseline models vs $\text {SSL-RE}_{sdp}$. All models are trained using SDP finetuning. SDP tokens used in finetuning are highlighted as green. In the first four examples, $\text {SSL-RE}_{sdp}$ can accurately captures the gold relations between entities, while in the last example self-training and RE-Ensemble performed better.

### In-context learning

The aim of this experiment is to assess the effect of utilizing Shortest Dependency Path (SDP) representation to boost the accuracy of Relation Extraction (RE) within the in-context-learning framework. To this end, we furnish the GPT-3 model with task-specific instructions and a few examples that illustrate the task at hand.

Recent research [11, 12] indicates that dynamically selecting in-context examples for each test instance, rather than employing a fixed set of in-context examples, results in notable improvements in GPT-3’s in-context learning. Taking inspiration from the approach outlined in [11], we implement a k-nearest neighbor (kNN) retrieval module to identify the most closely related examples from our constrained training dataset to serve as the in-context prompts for each test instance. During this process, we use the SDP representation as the basis for calculating the distance metric, which in turn determines the similarity between the test and training instances.

In our experiments, we allocate a training budget of 50 and, in each test, we contrast the efficacy of SDP-based nearest neighbor retrieval with random and fixed prompting. In the random prompting scenario, we arbitrarily select in-context examples from the training dataset for every test instance, while in the fixed prompt setting, we maintain a consistent set of examples across all test sets. We employ stratified sampling to ensure each relation type is represented in the prompt along with the task instruction. To carry out this task, we leverage the highly potent GPT-3 DaVinci engine. However, due to cost considerations associated with using GPT-3, we restrict our test set to a subsample of 200 examples for each experiment. For both fixed and random prompts, we repeat the experiments three times (keeping the test set constant but varying the in-context examples) to establish the reliability of our results.

As depicted in Table 6, the inclusion of SDP-based nearest neighbor retrieval in Drug-Drug Interaction (DDI) led to a considerable improvement in performance for both fixed and random prompts. A modest positive effect was observed for ChemProt, with an approximate increase of 1.9% in performance. However, no discernible improvement was recorded for Protein-Protein Interaction (PPI).

**Table 6:** Using SDP to retrieve NN in few-shot experiments versus random and fixed example selection in prompts in in-context-learning. $\text {SDP}_{nn}$ indicates using SDP to retrieve nearest neighbors. $\text {SSL-RE}_{sdp}$ indicates the semi-supervised performance on 50 training budget and tested on the same test set (200 examples)

	DDI			ChemProt			PPI		
	P	R	F1	P	R	F1	P	R	F1
$\text {SSL-RE}_{sdp}$	0.63	0.50	0.56	0.69	0.43	0.53	0.81	0.62	0.71
GPT3 (fixed)	0.45	0.37	0.40	**0.72**	0.40	0.51	**0.61**	**0.80**	**0.69**
GPT3 (random)	0.45	0.41	0.43	0.69	0.36	0.47	0.60	0.78	0.68
GPT3 ($\text {SDP}_{nn}$)	**0.51**	**0.50**	**0.50**	0.71	**0.41**	**0.52**	**0.61**	**0.80**	**0.69**

### Ablation study

#### Choice of representation on augmentation module

We investigate the impact of SDP on the label imputation. To this end, we performed experiments on different sequence representations to compute distance metric, and thereafter used the imputed labels to train the RE model. Note that we keep $\text {SUP-RE}_{sdp}$ architecture for the RE model training, and only changed the representation in the imputation. We use the following representations to extract from the last hidden state of the encoder $\mathcal {Q}$.

- CLS (L): is an aggregate representation of all the tokens in a sentence
- ent-avg (L): is the average embedding of the entities in a sentence
- ent-sdp-avg (L): is the average embedding of the entities and SDP tokens
- ENT (2L): is concatenation of the embeddings of two entities in a sentence
- ent-words-between (3L): is concatenation of the embeddings of the two entities along with the average representation of all the words between two entities
- $\text {SDP}_{\text {rep}}$ (3L): is our proposed representation in Eq. 5

As shown in Table 7, $\text {SDP}_{\text {rep}}$ representation results in overall better F1 score compared to other representations. By comparing the average performance of all representations across all datasets, we observed that $\text {SDP}_{\text {rep}}$ ranked highest achieving 0.39 average F1 score, ent-sdp-avg ranked second with 0.38 average F1, and CLS ranked lowest with 0.35 average F1.

The representation ent-words-between achieved second to the last (0.37 average F1), meaning adding unnecessary context does not help to find high-quality neighbor search.

**Table 7:** Impact of representation choice in augmentation module, and the resulting performance of RE model. We experimented with 200 labeling examples

	Dim	DDI	ChemProt	PPI
CLS	L	0.30	0.24	0.51
ent-avg	L	0.30	0.33	0.52
ent-sdp-avg	L	0.32	0.31	0.53
ENT	2L	0.30	0.33	0.51
ent-words-between	3L	0.32	0.28	0.52
$\text {SDP}_{\text {rep}}$	3L	**0.32**	**0.33**	**0.54**

#### Effectiveness of soft labels

To better understand the impact of soft label assignment in weak label imputation, Table 8 reports the performance against hard label assignment, where we only take the label with highest probability during training. We could see that soft labels improve the performance on DDI and ChemProt datasets by 13% and 8% in F1. There is no improvement over PPI dataset.

**Table 8:** Effectiveness of soft label assignment in three datasets using 200 training data

	DDI	ChemProt	PPI
$\text {SSL-RE}_{sdp}$ (hard label)	0.32	0.32	**0.52**
$\text {SSL-RE}_{sdp}$ (soft label)	**0.37**	**0.35**	0.51

## Discussion and limitation

Our study demonstrates the SDP’s linear scalability which is a critical factor for practical large-scale applications. In practical testing, SDP generation took only 8.86 seconds for 500 samples, while scaling to larger datasets, such as 2000 samples, necessitated a proportional increase in computation time to 34.05 seconds. This efficient preprocessing enables the model’s use in extensive literary corpora, such as PubMed abstracts, without imposing significant computational delays.

Our findings suggest that the integration of SDP with nearest neighbor enriches the model with nuanced syntactic and semantic context while carefully imputing pseudo labels. However, as dataset sizes grow, the method’s relative benefit may diminish due to stronger inherent patterns within the data. Nevertheless our approach offers a pragmatic and feasible solution for initial analyses, beneficial for users needing immediate insights without the complexity of larger models.

Moreover, the SDP representation can seamlessly augment the capabilities of existing off-the-shelf RE models, thereby enhancing their accuracy and reliability for comprehensive analysis.

We acknowledge certain limitations in our methodology. One limitation of our work is that we assume that unlabeled and labeled data are sampled from the same distribution. If the sampling of labeled data is biased, our label imputation approach may not work that well.

Second, our approach depends on the availability of a good dependency parser. This is a limitation if the proposed approach is used on rare languages or in very specialized domains. Third, all three of our datasets had a relatively small number of clearly delineated relation types. It would be important for future work to exploit the effectiveness of the proposed approach on data with a much larger number of relation types.

Fourth, our experiments were performed using the BERT encoder. While it is one of the first strong LLM models, we have recently witnessed the emergence of much stronger models such as GPT-4. It remains an open question if SDP representation could be helpful to those newer LLMs. There are two reasons we did not use GPT-4 or comparable models. First, most of those models are proprietary and inaccessible to researchers. The open-sourced versions are typically much weaker for multiple reasons. In addition, the state-of-the-art LLMs are also extremely large, and our lab did not have sufficient computational resources to support experimenting with those models.

## Conclusion

This study demonstrates the utility of Shortest Dependency Path (SDP) representations in supervised, semi-supervised, and in-context learning for low-resource biomedical relation extraction (RE). We introduced an innovative SDP-based representation, which we employed to compute the distance metric between RE instances. In addition, we proposed a new semi-supervised learning (SSL) algorithm tailored for biomedical RE. Comprehensive experimental assessments on three biomedical text datasets substantiate the effectiveness of SDP representation. Importantly, our proposed approaches are not tied to a specific neural network architecture and can be seamlessly integrated as a wrapper around existing and future RE models.

## Data Availability

The datasets used and/or analysed during the current study are available from the corresponding author on reasonable request.

## Abbreviations

BERT: Bidirectional encoder representations from transformers
GPT-3: Generative Pre-trained Transformer 3
RE: Relation Extraction
SciBERT: Science BERT
SDP: Shortest Dependency Path
SSL: Semi Supervised Learning

## Footnotes

1 DualRE. https://github.com/INK-USC/DualRE. Accessed 11 July 2023.

2 Student’s t-test. https://en.wikipedia.org/wiki/Student%27s_t-test. Accessed 11 July 2023
